# ACKNOWLEDGEMENTS TO REVIEWERS

**DOI:** 10.2174/1570159X2214240709123828

**Published:** 2024-07-09

   Bentham Science Publishers would like to thank and appreciate the co-operation from all reviewers for their constructive comments and feedback on the manuscripts submitted to “**Current Neuropharmacology**”. Their efforts have contributed greatly to the high quality and continuous growth of the journal. Given below is the list of reviewers who reviewed articles for the Journal during 2024:

Agata Copani (Italy)

Agnieszka Basta-Kaim (Poland)

Agorastos Agorastos (Greece)

Alasdairm Barr (Canada)

Aleksander Araszkiewicz (Poland)

Aleksey Zaitsev (Russian Federation)

Alice Câmara (Brazil)

Andrea Aguglia (Italy)

Andrius Baskys (USA)

Antoine Louveau (USA)

Antonio Del Casale (Italy)

Antonio Pisani (Italy)

Aurelio Freire M. Marco (Brazil)

Bertini Caterina (USA)

Bertozzi Giuseppe (Italy)

Bhavesh Variya (Canada)

Brondino Natascia (Italy)

Bruno Bonaz (USA)

Bryan Roth (USA)

Bryce Hruska (USA)

Celia Carlini (Brazil)

Chao Ma (USA)

Christopher Pittenger (USA)

Clarke Gerard (France)

Colin Barnstable (USA)

Conorr Murray (USA)

Cristian Falup-Pecurariu (USA)

Damian G. Zuloaga (USA)Daniele Caprioli (Italy)

David J. Brooks (USA)

Deniz Bagdas (USA)

Diego Centonze (Italy)

Domenico De Berardis (Italy)

Dominic Oliver (United Kingdom of Great Britain and Northern Ireland)

Fabiola Ribeiro (Brazil)

Fabrizio Stocchi (USA)

Felice Iasevoli (Italy)

Francesco Di Carlo (Italy)

Francesco Fornai (Italy)

Francesco Pontieri (Italy)

Gadelha Figueiredo Eberval (USA)

Gaelle E. Doucet (USA)

Gang Chen (China)

Giovanni Martinotti (Italy)

Giuseppe Caruso (Italy)

Giuseppe Gangarossa (France)

Goldstein Jorge (Argentina)

Gottardo G. Nicholas (Australia)

Graybiel M. Ann (USA)

Gustav Akk (USA)

Haiwei Xu (USA)

Ilse Smolders (Belgium)

Istvan Bokkon (USA)

Jaroslav Pejchal (Czech Republic)

Jeffrey M. Witkin (USA)

Jialin Zheng (USA)

Jianguo Feng (China)

Jolanta B. Zawilska (Poland)

José Nogueira-Machado (Brazil)

Kalyani Barve (India)

Ke Zhao (China)

Kenneth Blum (USA)

Lanxin Luo (China)

Laura Rizzi (Italy)

Loonen J.M. Anton (Netherlands)

Manuela Causo (USA)

Marco Atzori (USA)

Marco Avila-Rodriguez (Colombia)

Marco Fiore (Italy)

Marco Peviani (Italy)

Marco Scarselli (Italy)

Maria Luisa Barbaccia (Italy)

Marianne Amalric (France)

Marika Alborghetti (Italy)

Masahiko Watanabe (Japan)

Massimo Allegri (Switzerland)

Massimo Pasquini (Italy)

Mateusz Maciejczyk (USA)

Maurizio Muraca (USA)

Maurizio Popoli (Italy)

Maxime Assous (United Kingdom of Great Britain and Northern Ireland)

Milena Cannella (Italy)

Monica Butnariu (Romania)

Mstteo Caridi (Italy)

Murat Senturk (USA)

Nadine Attal (USA)

Natalia Stefanova (Russian Federation)

Ozal Beylerli (Russian Federation)

Paolo Calabresi (Italy)

Patrick Baril (France)

Peng Huang (USA)

Pengfei Yang (China)

Peter Kokol (Slovenia)

Pierangelo Geppetti (Italy)

Pierre Blanchet (USA)

Pradeep Bhide (USA)

Pragati Khare (India)

Prashant Tiwari (India)

Rainer Glaser (USA)

Rajeshwara Arya (India)

Robert Kumsta (Luxembourg)

Roberta Celli (Italy)

Rodolfo Cavalcanti Jose (Brazil)

Roland Brandt (USA)

Rui-Xian Guo (USA)

Santina Chiechio (Italy)

Sari Goldstein Ferber (Israel)

Serena Notartomaso (Italy)

Seth D. Norrholm (USA)

Sharon DeMorrow (USA)

Simona Pichini (Italy)

Sinan Guloksuz (USA)

Stefan Leucht (USA)

Stefania Bonaccorso (United Kingdom of Great Britain and Northern Ireland)

Stefania Chiappini (United Kingdom of Great Britain and Northern Ireland)

Stefano D'Errico (Italy)

Sten Grillner (Sweden)

Thomas Kuhn (USA)

Ubaldo Bonuccelli (USA)

Vadim Bolshakov (USA)

Vahid Reza Askari (The Islamic Republic of Iran)

Vincenzo Micale (Italy)

Vito de Novellis (Italy)

Vittorio Calabrese (Italy)

Wataru Araki (Japan)

Werner Treptow (Brazil)

William D. Hutchison (USA)

Xiao-Ping Wang (China)

Yu Stepanichev Mikhail (Russian Federation)

Zeljko Reiner (Croatia)

Zrinka Kovarik (USA)

